# Global sport and the challenges of political life

**DOI:** 10.3389/fspor.2023.1208264

**Published:** 2023-05-10

**Authors:** Simon C. Darnell

**Affiliations:** Faculty of Kinesiology & Physical Education, University of Toronto, Toronto, ON, Canada

**Keywords:** sports, policy, politics, law, global

## Politics, Policy and Law

Welcome to this special section of *Frontiers in Sports and Active Living* focused on Politics, Policy and Law. It is a challenging time for global sport. On the one hand, sport is arguably as popular and influential as it has ever been, with record-high levels of revenues and consumption, particularly in elite professional sport,^1^ as well as relatively high levels of participation in some contexts,^2^ despite the challenges of COVID-19. As a result, the influence of sport—socially, economically, and politically—around the world cannot be ignored. And yet sport is also confronted by and complicit in fundamental global challenges, like the climate crisis, military aggression, unprecedented levels of forced migration, and direct challenges to social democratic values and institutions. The 2022 FIFA World Cup in Qatar offers an illustrative example of such tensions, as hosting the sports mega-event in the Middle East for the first time laid bare many of the contradictions of global sport. On the one hand, Qatar 2022 was hugely popular, drawing in 1.5 billion viewers for the final match,^3^ and ostensibly expanding the global sporting community into a new region while creating opportunities for improved geo-political relations and understanding. On the other hand, the corruption connected to the bid process that led to Qatar's hosting^4^, its position as a petrostate amidst the climate crisis,^5^ and the blatant and tragic human rights abuses during the construction of the tournament's facilities^6^ all exposed the various political, policy and legal challenges connected to global sport.

This section of *Frontiers in Sports and Active Living* is designed to examine such issues head-on, through social science research that is theoretically robust, empirically grounded, internationally diverse and critically informed.

Chief among the issues to be examined in this section is the ongoing use of sport by states and other various polities in support of political aspirations, both domestically and in the international sphere. The view that sport is attractive to governments and states in advancing their own agendas is not new; indeed, scholars like Lincoln Allison called critical attention to such politics of sport nearly 40 years ago (1). In turn, this body of work began to highlight the changing ways that states use sport to advance their interests, moving beyond sport as a form of benign international goodwill and towards an appreciation of sport as a means of asserting and demonstrating prestige (2). Such perspectives, relevant today more than ever, are less concerned with *whether* the state (broadly defined) values or uses sport to achieve its political ends, and instead asks questions of *how*, *why* and to *what ends* the state/sport relationship exists in contemporary geo-political contexts? Again, Qatar is a useful example; as analysts like James Dorsey have argued, what the World Cup offered primarily to Qatar (a tiny nation with a small permanent population) was an opportunity, *via* soft power, to insulate itself from the military and geographic machinations that have seen its neighboring countries like Kuwait and Yemen suffer as the sites of proxy wars.^7^ Where and how else are similar geo-politics playing out in and through sport? This section invites critical responses to such queries.

Another site for critical analysis within this section is both related to the above yet also different. Namely, what are the ways in which governments treat sport in their domestic policies? Again, this is a research question with a rich history; scholars like Barrie Houlihan and Mick Green in the UK, Mike Sam in New Zealand, and Jean Harvey and Lucie Thibault in Canada (to name but a few) have all documented the various priorities (and therefore politics) that are embedded into state sport policy. It is also here that key sport policy tensions and debates emerge that are ripe for critical analysis. For example, is the primary purpose of sport policy to produce elite athletes, who bring prestige and pride to the nation (3)? Or should the purpose be to increase sport participation at grassroots or local levels, or among those groups typically excluded (4)? Or perhaps the best approach is a hybrid one, in which elite athletes offer demonstrations to the public of what is possible and in ways that encourage increased participation (5)? Of course, none of this is straight forward, automatic, or guaranteed. Scholars like Fred Coalter (6) have argued that sport policy may even be external, or epiphenomenal, to sport participation because it is the structural and distributive social practices that underpin equality/inequality—not policy documents—that determine whether people play sports or not. And as many industrial/post-industrial nations experience growing levels of income inequality and inflation, and witness the ongoing growth of the precarious classes and the gig economy (7) the questions of what is to be pursued through public sport policy and whose interests are being served, take on even further significance. Add to the mix that such policies and policy makers are now also tasked with responding to crises of abuse in sport,^8^ and addressing the relationship between sport and the climate crisis,^9^ and it is clear that sport policy makers and scholars are navigating a new context.

These latter policy issues in sport lead into the emerging field of sports and the law. Several recent developments in sport have increased the significance, importance and necessity of strong legal frameworks. The aforementioned issue of migrant worker rights in building stadiums for sports mega-events like the FIFA World Cup is a case in point. What laws or protections are needed for workers in global sport? What laws are possible? Workers’ legal rights are also just one such case. The issue of trans rights, and policies and practices of gender verification—or sex testing—in sport have come crashing to the fore recently. As world governing bodies produce new policies—like the World Athletic Federation's recently announced regulations which prohibit athletes who have gone through “male puberty” from participating in the female category^10^—rights-based legal challenges and criticism are sure to follow. It is in this milieu that strong scholarship is needed that can serve to contextualize, and where necessary criticize, the ethics, politics and veracity of such regulations.

Here, again, the issue of safe sport remains of critical importance. While national governments continue to explore and develop policies and regulations to ensure that sport is safe, particularly for elite athletes who are often placed in subordinate relations of power relative to coaches, trainers, and doctors, the legal aspects of these issues need attention as well. What legal responsibility does sport have to maintain safe, abuse-free spaces and events for athletes? Where, precisely, does responsibility lie? And how can it best be achieved? Again, strong social science scholarship has a clear opportunity to contribute to these important conversations and debates.

These are big issues, but the complexity and richness of this field of sport Politics, Policy and Law extends even further, given that sport also has long been a site in and through various actors have engaged in political activism and dissent. It is here that the question about social movements in and through sport is still germane [see (8)]. Even though the media attention afforded Colin Kaepernick's kneeling protest before National Football League games has somewhat faded, there are still important relationships between sport and social movements currently in play. The connections, for example, between the Black Lives Matter movement and professional leagues like the WNBA, raise important empirical and theoretical questions about the appetite (or lack thereof) amongst athletes, sports leagues, clubs, fans and sponsors for open political discussions in sport, or whether sport is an effective venue for social movement actors to promote their causes. Similarly, the ongoing activism by women athletes to achieve equal pay relative to their male counterparts has brought the politics of the feminist movement into the field of sport in a forceful way in recent years.^11^ This politicization of sports in relation to activism and social movements is a fruitful site for social science research, particularly given ongoing substantive and theoretical debates about the processes and practices of social movements themselves in the new millennium (9).

Which brings us in some respects, back to the “classic” issues of sport and politics discussed at the outset of this short article. Namely, what role do, can or should the world's international sporting bodies play in the “arena” of global politics and in relation to fundamental political values and goals of democracy, justice, sovereignty, peace and development? Political scientists, and their core questions, remain important here. If, for example, we are to accept the International Olympic Committee as a credible political actor, what are the implications, in both theoretical and empirical terms? When the United Nations Office on Sport for Development and Peace closed in 2017, it was ostensibly to reduce the potential duplication between activities of the UN and the humanitarian work being done by the IOC.^12^ Historians and critics of the IOC, however, might suggest that while the IOC claims its support for international development and peace, its primary goal is nearly always to maintain an ascendent position within the international sporting hierarchy (10). From this perspective, claims to sport as a means of achieving international development and peace offer but the latest and best avenue through which the IOC can assert itself.

This, in turns, (re)raises classic political theory questions, such as the difference between a realist and idealist approach. For the realist, politics is firmly about power, and power over others, whereas the idealist perspective leaves some room for politics to be about “genuine attempts to create or maintain an international order based on shared values” (11). Which of these most accurately describes the current politics, policies and practices of sport organizations like the International Olympic Committee, particularly recognizing that such tensions are never zero-sum but more reflective of a continuum or values that shift and change in context and over time (11)? Amidst such complexity, our field needs scholars to enter such debates and to offer assessments supported by theoretical force and empirical rigour.

The stakes of this research agenda are high. The imminent threat of the climate crisis is front and centre, and sports organizations and stakeholders are, it must be said, rather late to the party, and have even, in some cases, abdicated their ethical responsibility by turning to the greenwashing of sport (12). But even beyond the existential threat to sport (and life) that the changing climate presents, questions remain as to what values and ethics (and their associated politics) are to be celebrated and upheld within sport itself, which stakeholders are responsible for this, and by and through which legal, political or social processes can they be promoted and disseminated? While we may welcome the idea that powerful sporting organizations like the IOC and FIFA could be forces for sustainable development and peace on a global scale, their track records,—which include sportswashing, the displacement of marginalized populations, quashing of activism and dissent, complicity in environmental destruction, and participation in bribery and corruption—should give all critical analysts pause. Amidst this high-stakes contestability, this special section of *Frontiers in Sports and Active Living* focused on Politics, Policy and Law offers an opportunity for scholars to contribute to making a difference in the world of global sport.

## Footnotes

^1^ https://www.forbes.com/sites/maurybrown/2023/01/10/mlb-sets-new-revenue-record-exceeding-108-billion-for-2022/?sh=29568c2277ee

^2^ https://www.sportsbusinessjournal.com/Journal/Issues/2022/06/13/Portfolio/Research.aspx

^3^ https://www.sportspromedia.com/news/qatar-2022-fifa-world-cup-final-argentina-france-viewers-engagement/?zephr_sso_ott=R95OsZ

^4^ https://www.nytimes.com/2020/04/06/sports/soccer/qatar-and-russia-bribery-world-cup-fifa.html

^5^ https://www.theatlantic.com/international/archive/2022/11/qatar-hosting-fifa-world-cup-soccer/672171/

^6^ https://www.hrw.org/tag/fifa-world-cup-qatar-2022

^7^ https://www.huffpost.com/entry/qatar-workers_b_4294207

^8^ https://torontosun.com/sports/safe-sport-crisis-shows-canadian-sport-system-needs-maintenance-if-not-total-overhaul

^9^https://www.euronews.com/green/2023/03/04/sport-is-both-a-climate-victim-and-villain-these-champions-show-theres-another-way#:∼:text=Sport%20is%20both%20a%20'victim'%20and%20contributor%20to%20climate%20change&text=Unlike%20many%20other%20industries%2C%20however,due%20to%20the%20excessive%20heat.

^10^ https://www.cnn.com/2023/03/23/sport/world-athletics-transgender-ruling-spt-intl/index.html

^11^ https://www.theguardian.com/football/2022/dec/16/uswnt-equal-pay-settlement-case

^12^ https://www.sportanddev.org/latest/news/quiet-demise-unosdp-where-do-we-go-here#:∼:text=%E2%80%9CThe%20Secretary%2DGeneral%20has%20agreed,and%20Peace%20(UNOSDP).%E2%80%9D
